# Standardization of *G. mellonella* Larvae to Provide Reliable and Reproducible Results in the Study of Fungal Pathogens

**DOI:** 10.3390/jof4030108

**Published:** 2018-09-06

**Authors:** Olivia L. Champion, Richard W. Titball, Steven Bates

**Affiliations:** 1 Biosystems Technology Ltd., 1 Colleton Crescent, Exeter EX2 4DG, UK; o.champion@biosystemstechnology.com; 2 College of Life and Environmental Science, University of Exeter, Stocker Road, Exeter EX4 4QD, UK; s.bates@exeter.ac.uk

**Keywords:** *Galleria mellonella*, infection model, fungi, genome, pathological score, end point

## Abstract

In the past decade, *Galleria mellonella* (wax moth) larvae have become widely used as a non-mammalian infection model. However, the full potential of this infection model has yet to be realised, limited by the variable quality of larvae used and the lack of standardised procedures. Here, we review larvae suitable for research, protocols for dosing larvae, and methods for scoring illness in larvae infected with fungal pathogens. The development of standardised protocols for carrying out our experimental work will allow high throughput screens to be developed, changing the way in which we evaluate panels of mutants and strains. It will also enable the in vivo screening of potential antimicrobials at an earlier stage in the research and development cycle.

## 1. Introduction

The economy, ease of maintenance, and ethical acceptability has led to the widespread adoption of *Galleria mellonella* (wax moth) larvae, as a non-mammalian infection model. The larvae can be incubated at 37 °C, allowing the expression of temperature-regulated virulence genes. Moreover, a defined infection site and the ability to challenge larvae with exact doses of fungi allow the 50% lethal dose (LD_50_) to be calculated. This allows the virulence of mutants, or the efficacy of antifungal compounds, to be compared and provides a major advantage over many other non-mammalian infection models (Table 1). The innate immune system of *G. mellonella* shares many similarities with the innate system of mammals [1]. Central to insect immunity are specialized phagocytic cells (hemocytes). Like mammalian neutrophils, they show lectin-mediated phagocytosis of microorganisms and kill via a respiratory burst mediated by NADPH oxidase [1]. Hemocytes display Toll-like receptors and binding activates antimicrobial peptide production via an NFκB-like signalling pathway [1]. The similarities between neutrophils and hemocytes allow the complex interplay between *G. mellonella* and the pathogen to be captured in a way that is not possible in cell culture infection systems. It is likely a combination of these features which has led to the widespread adoption of *G. mellonella* larvae as a model for infections caused by a wide range of fungi [2] including *Aspergillus* [3,4], *Candida* [5,6,7,8,9,10,11,12,13,14], and *Cryptococcus* species [15,16,17], although notably, the larvae are reported to be resistant to *Pneumoncystis murina* infection [18]. For some fungal pathogens, *G. mellonella* larvae are now becoming the infection model of choice, with over 115 publications to date using this model with *Candida albicans*. 

However, we believe that the full potential of the *G. mellonella* infection model has yet to be realised, held back by the variable quality of larvae used and the lack of standardised procedures for dosing larvae and recording morbidity and mortality. The aim of this review is to address the extent to which these problems have been resolved, and to identify future work needed to provide a robust, reliable, and consistent infection model. 

## 2. Reported Variability of Fungal Infection Models

As would be expected in an animal model of infection, variability is clearly apparent in the outcome data reported. The lack of standardised procedures for conducting these studies is thought to be a key factor contributing to the level of variability demonstrated, and this variability can prevent the direct comparison of published studies. The methodologies employed in these studies are broadly similar, but can display key differences in the preparation of inocula, injection volume, source and handling of larvae, and experimental conditions such as temperature. For example, published studies utilising the model to assess the virulence of *Candida albicans* mutants have reported using inoculum levels ranging almost one hundred fold (6 × 10^4^ to 5 × 10^6^ cells/larva). Furthermore, even when relatively similar procedures appear to have been employed, substantial variation can still be seen in the results published. For example, two recently published studies in *C. albicans* [19,20], utilising comparable wild type strains at the same inoculum level, reported mortality at five days to be either 20% or 60%. Another study measuring the virulence of 51 *C. albicans* transcription factor mutants in *G. mellonella* larvae reported only a 45% correlation between the results from replicate experiments, which they attributed to variability of the larvae [21]. Issues such as these may potentially be overcome through the standardization of assay protocols, plus the introduction of well-characterised *G. mellonella* lines.

## 3. Standardization of *G. mellonella* Larvae

Tsai et al. [22] have previously identified the lack of standardised *G. mellonella* larvae as a significant barrier to the wider adoption of this model for bacterial pathogens. For many years, *G. mellonella* larvae have been commercially available as food for captive reptiles and birds or as fishing bait, and larvae bred for these purposes have been widely used in research. These larvae are not age- or weight-defined, and have been bred, reared, and maintained under differing conditions. Age, feeding status, and physical handling of larvae have all been reported to have a significant impact on the susceptibility of the larvae to infection [23,24]. Furthermore, the larvae may contain antibiotic and hormone residues [25]. This can result in inconsistent responses of larvae to infection, possibly reflecting altered metabolism in the larvae [26]. To address these problems, standardised *G. mellonella* larvae (TruLarv™) are now available (www.BioSystemsTechnology.com). These larvae are purpose bred for research without antibiotics or hormones added to feedstuff. They are age and weight defined and the cuticle of the larvae is decontaminated, reducing the problem of infections in control animals injected with PBS. The use of these larvae as an infection model, in place of pet-food grade larvae, has been seen to have a major impact on the consistency and reproducibility of experiments with bacterial pathogens [27,28], and may also reduce the level of variation seen with fungal pathogens.

## 4. Standardization of Challenge and Dosing 

The most common method of infection is by injection of the larvae. This allows precise doses of a fungal pathogen to be given and consequently it is possible to calculate the LD_50_. There is also some interest in challenging larvae by the oral route. However, the larvae that are commercially available are fifth or sixth instar stage, and they feed little during this final phase before pupation. Therefore, oral dosing can only be achieved by using earlier instar stage larvae, or by oral gavage. These different dosing routes are reviewed below.

### 4.1. Subcutaneous Microinjection

Methods vary between laboratories, but commonly larvae are injected with 10 µL innocula (up to 40 µL innocula have also been used [5,21]) by sub cutaneous micro-injection into a defined site (often a proleg), using either a Hamilton or fine insulin syringe [29,30]. The exact type of needle used for injection needs to be considered in the context of the volume delivered. For example, insulin syringes have 10 µL increments, meaning that there may be error in delivering exactly 10 µL. The most precise delivery of 10 µL necessitates the use of a 10 µL Hamilton syringe. In some laboratories, larvae are immobilized between the operator's fingers and the needle inserted into the insect’s proleg, lifting the needle away from the operator with the insect attached before pushing the plunger on the syringe [31]. To reduce the risk of needle stick injury, a range of safety procedures have been developed by laboratories, including immobilising larvae over a pipette tip fixed to filter paper, the use of a stab-proof glove during injections [32], and a restraint device comprised of sponge and a bulldog clip termed the “Galleria grabber” [30].

Infected larvae may be incubated at temperatures ranging from 15 °C to 37 °C, as required. The ability to incubate larvae at 37 °C facilitates studies involving temperature-regulated virulence genes. PBS and uninfected controls are included in studies to ensure that larval death is not a result of trauma due to the injection. Groups of ten larvae are generally used in an experiment, with two or three experimental replicates providing large data sets for statistical analyses [29,33,34,35].

### 4.2. Feeding Larvae

To mimic the physiological route of natural exposure to microbes, *G. mellonella* have sometimes been fed microbes in their diet. Variations of this method have been reported, for example, Freitak et al. [36] fed third instar *G. mellonella* larvae a standard wax moth diet drenched with LB broth for control groups, or mixtures of microbial cultures in test groups [36]. Similarly, Chertkova et al. [37] used the oral route of infection. Following oral infection of *G. mellonella* larvae with combinations of microbes, the concentration of dopamine was measured at different time points in the haemolymph [37]. In this study, the oral inoculation of fourth instar larvae was performed after mixing microorganisms with artificial medium. Control groups were fed on artificial medium mixed with saline. 

### 4.3. Oral Gavage

Forced feeding of *G. mellonella* larvae has been reported in microbial infection studies [38,39]. To administer a suspension of microbes, oral gavage is required, in which a blunted microinjector syringe is gently inserted into the mouth piece of final instar larvae and 20 µL inoculum is delivered [40]. 

## 5. Standardised Scoring 

Early studies using *G. mellonella* as an infection model scored larval death as the endpoint, typically measured as the ability of larvae to move or respond to physical stimuli with a pipette tip. The use of a well-defined endpoint allows calculation of the LD_50_. A modification of this approach is to calculate the “virulence index” based on the time to death of 50% of the cohort, but normalised to the time to death of the wild type and expressed as a log value [14,41,42]. By calculating the virulence index, it is possible to compare data between laboratories more simply. However, the monitoring of larval death in this way at frequent intervals can be very time consuming, and therefore it is usual to score the larvae at 12 or 24 h intervals. Whilst this can reduce the workload, it can also result in fine differences in the time to death being missed.

**Figure 1:**
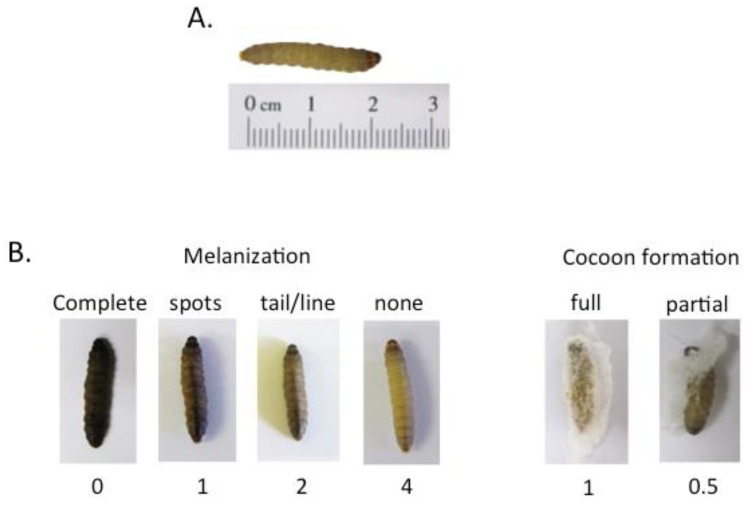
Changes in the appearance of *G. mellonella* larvae after infection (**B**) compared to healthy final instar stage larvae (**A**). Reproduced from [22] with the permission of the authors. Progressive melanisation of larvae is shown from right (none) to left (complete).

Other ways of measuring infection include recording the progressive melanisation of larvae (Figure 1), the direct enumeration of pathogens within body tissues, and histology on infected larvae. These methods all have different benefits and drawbacks. In our experience, the degree of melanisation is dependent on the infecting pathogen. Some pathogens cause profound and uniform melanisation, whilst others cause more subtle colour changes which can be difficult to interpret. The enumeration of pathogen load in tissues, or histology, both require the culling and processing of larvae which can be time consuming. Pathogen load at given time-points may also be calculated by homogenising the larvae and enumerating fungi after plating onto suitable media. The fungal load in larval compartments such as the cadaver, hemolymph, and hemocoel, may be established by draining hemolymph from infected larvae and using centrifugation to separate hemocytes from the hemolymph. Finally, the inability to form a silk cocoon by pupating larvae (Figure 1) indicates poor health.

Against this background, a pathological scoring system (Table 2) has been proposed by Loh et al. [43]. This system allows subtle differences in larval health to be assessed based on their appearance (Figure 1). It also facilitates greater reproducibility, and the comparison of data, between different laboratories. However, this scoring system still relies on time consuming checks. In the future, automated real-time imaging of larvae, possibly using the criteria outlined in Table 1, would open up opportunities for high throughput screens to be devised. 

## 6. High Throughput Screens

The low cost and ease with which *G. mellonella* larvae can be injected have prompted suggestions that this infection model could be used for high-throughput screening, either to identify virulence genes or to screen antifungal drugs. An important issue is how reliably these screens in *G. mellonella* larvae predict behaviour in mammals. There are some pivotal experiments which shed light on this question. In a study with *C. neoformans*, 46 of 66 mutants found to be attenuated in *G. mellonella* larvae (70%) had previously been shown to be attenuated in mice [44]. In contrast, the same study found that only 29% of mutants found to be attenuated in *C. elegans* were attenuated in mice [44]. The authors concluded that the increased discriminating power of *G. mellonella* likely reflects the greater similarity of the immune system to the mammalian immune system. In another study, the virulence of 18 *C. albicans* mutants was compared in mice and in *G. mellonella* larvae, and there was found to be a 50% correlation between the two lists of attenuated mutants [21]. However, mutants with strongly attenuated phenotypes in mice were much better predicted in *G. mellonella* larvae. This finding suggests that *G. mellonella* larvae are well suited to identifying gene products which play the most important roles in infection, and which are in any case likely to be the most attractive targets for interventions and exploitation. These findings add significant weight to the argument that high throughput screening using *G. mellonella* larvae provides meaningful results.

### 6.1. High Throughput Screening of Mutant Libraries

It is feasible to test large panels (up to 264) of fungal mutants individually in larvae [14,44,45]. However, to improve the statistical power of these studies, and because of the variability of responses of pet-shop larvae, groups of up to 50 larvae have been reported to be required for each mutant tested [14]. Clearly, the need to work with large cohorts of larvae would limit the number of mutants that can be tested simultaneously, and therefore the potential to carry out high throughput screens. Another approach would involve the simultaneous screening of mixtures of mutants or strains. By marking the different genotypes, for example, each with a unique DNA tag, it is possible to track the individual mutants within the population. Therefore, by tracking the abundance of DNA tags, genotypes which are more or less competitive (i.e., more or less virulent) in vivo are revealed. This approach has previously been widely used with bacterial and fungal pathogens in mammalian models of disease [46,47,48,49,50,51]. A major advantage of this high-throughput screen is the ability to test large groups (thousands or tens of thousands) of mutants or strains simultaneously in a single animal. Therefore, relatively small numbers of larvae can be used. But there is also a major disadvantage in that the virulence phenotype may not be revealed when pools of mutants are tested [21]. In some cases, this is because “cheater” mutants are able to survive and grow because other members of the population provide the necessary factors masking the virulence defect [21]. Notwithstanding this concern, this approach has been used to simultaneously compare the virulence of a panel of 4110 mutants of Saccharomyces cerevisiae in *G. mellonella* larvae [52]. This study found that genes involved in cell wall integrity, mitochondrial function, and tyrosine metabolism play key roles in disease. 

### 6.2. Screening for Antifungal Agents

Previous studies have found a good correlation between the efficacy of antifungal drugs in mammalian infection models and in *G. mellonella* models of disease [2]. *G. mellonella* larvae might enable high-throughput drug screening in two ways. One relies on an initial in vitro screen of compounds, followed by the testing of selected compound(s) in larvae. This could allow the testing of compounds in larvae at an earlier stage and on a larger scale than would be possible if mammals were used. This approach has already been used to identify novel antimicrobials [53,54,55,56,57,58,59,60,61,62,63,64,65,66,67,68,69,70].

Alternatively, compounds could be tested individually in *G. mellonella* larvae at the earliest possible stage. This approach has the advantage that the most promising leads are identified, but the disadvantage that compounds with low bioavailability or low stability in vivo may be missed. Many early stage compounds are poorly soluble in water, and testing may require the drug to be dissolved in a solvent such as dimethyl sulfoxide (DMSO), ethanol, or methanol. In our hands, even small (10 µL) volumes of these solvents are lethal to *G. mellonella* larvae unless diluted to 20% (*v*/*v*) DMSO or 30% (*v*/*v*) ethanol [71]. Other limitations of this approach are the number of larvae that can be dosed with compound and pathogen, the feasibility of keeping large cohorts of larvae, and the feasibility of recording morbidity and mortality or large groups of larvae. 

There are reports of the parallel screening of relatively small panels (<30) of antimicrobial compounds for activity towards microorganisms [72,73]. However, there has been little reported progress in developing *G. mellonella* larvae for screening larger panels of compounds in high-throughput screens. In part, this may reflect the variability of responses seen between pet-food grade larvae. For example, in one study, test groups of 30 larvae were used per test compound [73]. The availability of larvae that behave consistently might now allow screening with smaller groups of larvae and open new opportunities for high throughput screening.

## 7. Discussion

The larvae of the wax moth *G. mellonella* are undoubtedly becoming a popular model for studying microbial virulence and treatment options, as evidenced through both the expanding range of pathogens tested in the system and the growing number of reports utilising the model. The model is increasingly being accepted as an alternative to mammalian infection models, which are subject to greater ethical and logistical constraints. In addition, it displays key advantages over other invertebrate models, such as its ability to be maintained at human body temperature, and the ease of handling and delivery of a precise infective dose. Furthermore, these attributes allow large numbers of larvae to be infected, therefore facilitating its use for the large-scale screening of virulence factors or antimicrobial activities of candidate drugs. However, this model does suffer from some limitations and disadvantages, mainly surrounding the lack of standardised protocols and a standard well-characterised *G. mellonella* strain (Table 3).

There are a range of factors to consider when standardising the handling of wax moth larvae, including their age and size, availability of food [24], the physical stress associated with transportation [23], and incubation temperature and storage time [24]. Larvae from breeders supplying pet shops have also been seen to carry residual levels of antibiotics and hormones [25], and these have been suggested to have an impact on the variability seen in the model with bacterial pathogens and may also lead to an altered outcome with fungal pathogens. All of these factors can impact the response of larvae to infection, and therefore impact the level of variability seen with the model. Until recently, there has also been the lack of a standardised *G. mellonella* strain, with most reports utilising larvae from commercial pet-shop breeders, or occasionally through research groups maintaining their own colony. Therefore, in addition to differences in rearing and maintenance, strain differences may also impact the variability seen in the assay. Recently, however, the first commercial supplier of “research grade” larvae (TruLarv™) has been established, and early reports suggest that their use has lowered the level of variability seen with bacterial pathogens [28]. Finally, the wax moth model still lacks an annotated genome and the genetic tractability available in other insect models. However, the first report of a *G. mellonella* genome has recently been released [74], and this will hopefully now lead to its annotation to support the initial transcriptomics analysis of immune system genes [75]. This resource could then facilitate the development of molecular tools in *G. mellonella* in order to further our ability to use this increasingly popular model to dissect the host-pathogen interaction.

## Figures and Tables

**Table 1:** Comparison of alternative infection models.

Model	Whole Animal Model	Use at 37 °C	Precise Dosing	Immune System	Cost of Maintenance by User	Regulated Use in the UK
Monolayer cell cultures	no	yes	yes	no	medium	no
3D cell cultures	no	yes	yes	no	high	no
*Caenorhabditis elegans* (nematode)	yes	no	no	yes	low	no
*Panagrellus redivivus* (nematode)	yes	yes	no	yes	low	no
Zebra fish (and embryos)	yes	no	yes	yes	high	yes (fish and older embryos)
*Drosophila melanogaster* (fruit fly)	yes	yes	no	yes	low	no
*G. mellonella*	yes	yes	yes	yes	low	no
*Manduca sexta* (tobacco hornworm)	yes	yes	yes	yes	low	regulated as a crop pest.

**Table 2:** The *G. mellonella* health index scoring system [43].

Category	Description	Score
activity	no movement	0
	minimal movement on stimulation	1
	move when stimulated	2
	move without stimulation	3
cocoon formation	no cocoon	0
	partial cocoon	0.5
	full cocoon	1
melanisation	black larvae	0
	black spots on brown larvae	1
	≥3 spots on beige larvae	2
	<3 spots on beige larvae	3
	no melanisation	4
survival	dead	0
	alive	2

**Table 3:** Approaches to standardizing experiments using *G. mellonella* larvae.

Model Limitations	Consequences	Solution
Differences in age, weight and health status of larvae	Lack of reproducibility between experiments	Use age and weight defined larvae
Antibiotic and hormone residues	Lack of reproducibility between experiments. May distort the results of tests of antimicrobial efficacy	Use larvae bred without the use of antibiotics or hormones
Genetic diversity of *G. mellonella*	Lack of reproducibility between experiments	Use inbred breeding colony
Larvae have a surface flora of pathogenic microorganisms	Deaths in controls when injected with PBS	Surface decontaminate larvae
Larvae normally available do not feed	Difficult to dose orally with pathogens or chemicals	Use early instar stage larvae or Oral gavage
Scoring or morbidity or mortality can be subjective	End points are not well defined	Use Heath Index Scoring System
Dosing involves injection of small volumes	Dose of pathogen given is not precisely determined	Use Hamilton syringes
High throughput screening is limited by operator ability to inject large numbers of larvae	Screens are size limited	Reduce cohort size using standardised larvae or Develop automated screens

## References

[1] Browne N., Heelan M., Kavanagh K. (2013). An analysis of the structural and functional similarities of insect hemocytes and mammalian phagocytes. Virulence.

[2] Binder U., Maurer E., Lass-Florl C. (2016). Galleria mellonella: An invertebrate model to study pathogenicity in correctly defined fungal species. Fungal Biol..

[3] Jackson J.C., Higgins L.A., Lin X. (2009). Conidiation color mutants of *Aspergillus fumigatus* are highly pathogenic to the heterologous insect host *Galleria mellonella*. PLoS ONE.

[4] Geissel B., Penka M., Neubauer M., Wagener J. (2017). The ER-mitochondria encounter structure contributes to hyphal growth, mitochondrial morphology and virulence of the pathogenic mold *Aspergillus fumigatus*. Int. J. Med. Microbiol..

[5] Cotter G., Doyle S., Kavanagh K. (2000). Development of an insect model for the in vivo pathogenicity testing of yeasts. FEMS Immunol. Med. Microbiol..

[6] Brennan M., Thomas D.Y., Whiteway M., Kavanagh K. (2002). Correlation between virulence of *Candida albicans* mutants in mice and *Galleria mellonella* larvae. FEMS Immunol. Med. Microbiol..

[7] Borman A.M., Szekely A., Johnson E.M. (2016). Comparative pathogenicity of United Kingdom isolates of the emerging pathogen *Candida auris* and other key pathogenic *Candida* species. mSphere.

[8] Junqueira J.C., Fuchs B.B., Muhammed M., Coleman J.J., Suleiman J.M., Vilela S.F., Costa A.C., Rasteiro V.M., Jorge A.O., Mylonakis E. (2011). Oral *Candida albicans* isolates from HIV-positive individuals have similar in vitro biofilm-forming ability and pathogenicity as invasive *Candida* isolates. BMC Microbiol..

[9] Borghi E., Andreoni S., Cirasola D., Ricucci V., Sciota R., Morace G. (2014). Antifungal resistance does not necessarily affect *Candida glabrata* fitness. J. Chemother..

[10] Gago S., Garcia-Rodas R., Cuesta I., Mellado E., Alastruey-Izquierdo A. (2014). *Candida parapsilosis*, *Candida orthopsilosis*, and *Candida metapsilosis* virulence in the non-conventional host *Galleria mellonella*. Virulence.

[11] Souza A.C., Fuchs B.B., Pinhati H.M., Siqueira R.A., Hagen F., Meis J.F., Mylonakis E. (2015). *Candida parapsilosis* resistance to fluconazole: Molecular mechanisms and in vivo impact in infected *Galleria mellonella* Larvae. Antimicrob. Agents Chemother..

[12] Mesa-Arango A.C., Forastiero A., Bernal-Martinez L., Cuenca-Estrella M., Mellado E., Zaragoza O. (2013). The non-mammalian host *Galleria mellonella* can be used to study the virulence of the fungal pathogen *Candida tropicalis* and the efficacy of antifungal drugs during infection by this pathogenic yeast. Med. Mycol..

[13] Moralez A.T., Perini H.F., Furlaneto-Maia L., Almeida R.S., Panagio L.A., Furlaneto M.C. (2016). Phenotypic switching of *Candida tropicalis* is associated with cell damage in epithelial cells and virulence in *Galleria mellonella* model. Virulence.

[14] Ames L., Duxbury S., Pawlowska B., Ho H.L., Haynes K., Bates S. (2017). *Galleria mellonella* as a host model to study *Candida glabrata* virulence and antifungal efficacy. Virulence.

[15] Gago S., Serrano C., Alastruey-Izquierdo A. (2017). Molecular identification, antifungal resistance and virulence of *Cryptococcus neoformans* and *Cryptococcus deneoformans* isolated in Seville, Spain. Mycoses.

[16] Firacative C., Duan S., Meyer W. (2014). *Galleria mellonella* model identifies highly virulent strains among all major molecular types of *Cryptococcus gattii*. PLoS ONE.

[17] Mylonakis E., Moreno R., El Khoury J.B., Idnurm A., Heitman J., Calderwood S.B., Ausubel F.M., Diener A. (2005). *Galleria mellonella* as a model system to study *Cryptococcus neoformans* pathogenesis. Infect. Immun..

[18] Fuchs B.B., Bishop L.R., Kovacs J.A., Mylonakis E. (2011). *Galleria mellonella* are resistant to *Pneumocystis murina* infection. Mycopathologia.

[19] Bohovych I., Kastora S., Christianson S., Topil D., Kim H., Fangman T., Zhou Y.J., Barrientos A., Lee J., Brown A.J. (2016). Oma1 Links mitochondrial protein quality control and TOR signaling to modulate physiological plasticity and cellular stress responses. Mol. Cell. Biol..

[20] Patterson M.J., McKenzie C.G., Smith D.A., da Silva Dantas A., Sherston S., Veal E.A., Morgan B.A., MacCallum D.M., Erwig L.P., Quinn J. (2013). Ybp1 and Gpx3 signaling in *Candida albicans* govern hydrogen peroxide-induced oxidation of the Cap1 transcription factor and macrophage escape. Antioxid. Redox Signal..

[21] Amorim-Vaz S., Delarze E., Ischer F., Sanglard D., Coste A.T. (2015). Examining the virulence of *Candida albicans* transcription factor mutants using *Galleria mellonella* and mouse infection models. Front. Microbiol..

[22] Tsai C.J., Loh J.M., Proft T. (2016). *Galleria mellonella* infection models for the study of bacterial diseases and for antimicrobial drug testing. Virulence.

[23] Mowlds P., Barron A., Kavanagh K. (2008). Physical stress primes the immune response of *Galleria mellonella* larvae to infection by *Candida albicans*. Microbes Infect..

[24] Banville N., Browne N., Kavanagh K. (2012). Effect of nutrient deprivation on the susceptibility of *Galleria mellonella* larvae to infection. Virulence.

[25] Buyukguzel E., Kalender Y. (2007). Penicillin-induced oxidative stress: Effects on antioxidative response of midgut tissues in instars of *Galleria mellonella*. J. Econ. Entomol..

[26] Browne N., Surlis C., Maher A., Gallagher C., Carolan J.C., Clynes M., Kavanagh K. (2015). Prolonged pre-incubation increases the susceptibility of *Galleria mellonella* larvae to bacterial and fungal infection. Virulence.

[27] Wagley S., Borne R., Harrison J., Baker-Austin C., Ottaviani D., Leoni F., Vuddhakul V., Titball R.W. (2018). *Galleria mellonella* as an infection model to investigate virulence of *Vibrio parahaemolyticus*. Virulence.

[28] Wagley S., Champion O.L., Titball R.W. Case Study: Identification of Virulence Genes. https://biosystemstechnology.com/applications.

[29] Champion O.L., Karlyshev A.V., Senior N.J., Woodward M., La Ragione R., Howard S.L., Wren B.W., Titball R.W. (2010). Insect infection model for *Campylobacter jejuni* reveals that O-methyl phosphoramidate has insecticidal activity. J. Infect. Dis..

[30] Dalton J.P., Uy B., Swift S., Wiles S. (2017). A novel restraint device for injection of *Galleria mellonella* larvae that minimizes the risk of accidental operator needle stick injury. Front. Cell. Infect. Microbiol..

[31] Fuchs B.B., O'Brien E., Khoury J.B., Mylonakis E. (2010). Methods for using *Galleria mellonella* as a model host to study fungal pathogenesis. Virulence.

[32] Harding C.R., Schroeder G.N., Collins J.W., Frankel G. (2013). Use of *Galleria mellonella* as a model organism to study *Legionella pneumophila* infection. J. Vis. Exp..

[33] Champion O.L., Cooper I.A., James S.L., Ford D., Karlyshev A., Wren B.W., Duffield M., Oyston P.C., Titball R.W. (2009). *Galleria mellonella* as an alternative infection model for *Yersinia* pseudotuberculosis. Microbiology.

[34] Mukherjee K., Altincicek B., Hain T., Domann E., Vilcinskas A., Chakraborty T. (2010). *Galleria mellonella* as a model system for studying *Listeria* pathogenesis. Appl. Environ. Microbiol..

[35] Seed K.D., Dennis J.J. (2008). Development of *Galleria mellonella* as an alternative infection model for the *Burkholderia cepacia* complex. Infect. Immun..

[36] Freitak D., Schmidtberg H., Dickel F., Lochnit G., Vogel H., Vilcinskas A. (2014). The maternal transfer of bacteria can mediate trans-generational immune priming in insects. Virulence.

[37] Chertkova E.A., Grizanova E.V., Dubovskiy I.M. (2018). Bacterial and fungal infections induce bursts of dopamine in the haemolymph of the Colorado potato beetle *Leptinotarsa decemlineata* and greater wax moth *Galleria mellonella*. J. Invertebr. Pathol..

[38] Fedhila S., Buisson C., Dussurget O., Serror P., Glomski I.J., Liehl P., Lereclus D., Nielsen-LeRoux C. (2010). Comparative analysis of the virulence of invertebrate and mammalian pathogenic bacteria in the oral insect infection model *Galleria mellonella*. J. Invertebr. Pathol..

[39] Mukherjee K., Raju R., Fischer R., Vilcinskas A. (2013). *Galleria mellonella* as a model host to study gut microbe homeostasis and brain infection by the human pathogen *Listeria* monocytogenes. Adv. Biochem. Eng. Biotechnol..

[40] Maguire R., Kunc M., Hyrsl P., Kavanagh K. (2017). Caffeine administration alters the behaviour and development of *Galleria mellonella* larvae. Neurotoxicol. Teratol..

[41] Brunke S., Quintin J., Kasper L., Jacobsen I.D., Richter M.E., Hiller E., Schwarzmuller T., d‘Enfert C., Kuchler K., Rupp S. (2015). Of mice, flies–and men? Comparing fungal infection models for large-scale screening efforts. Dis. Model. Mech..

[42] Glittenberg M.T., Silas S., MacCallum D.M., Gow N.A., Ligoxygakis P. (2011). Wild-type Drosophila melanogaster as an alternative model system for investigating the pathogenicity of *Candida albicans*. Dis. Model. Mech..

[43] Loh J.M., Adenwalla N., Wiles S., Proft T. (2013). *Galleria mellonella* larvae as an infection model for group *A streptococcus*. Virulence.

[44] Desalermos A., Tan X., Rajamuthiah R., Arvanitis M., Wang Y., Li D., Kourkoumpetis T.K., Fuchs B.B., Mylonakis E. (2015). A multi-host approach for the systematic analysis of virulence factors in *Cryptococcus neoformans*. J. Infect. Dis..

[45] Lee K.T., So Y.S., Yang D.H., Jung K.W., Choi J., Lee D.G., Kwon H., Jang J., Wang L.L., Cha S. (2016). Systematic functional analysis of kinases in the fungal pathogen *Cryptococcus neoformans*. Nat. Commun..

[46] Van Opijnen T., Camilli A. (2013). Transposon insertion sequencing: A new tool for systems-level analysis of microorganisms. Nat. Rev. Microbiol..

[47] Barquist L., Boinett C.J., Cain A.K. (2013). Approaches to querying bacterial genomes with transposon-insertion sequencing. RNA Biol..

[48] McAdam P.R., Richardson E.J., Fitzgerald J.R. (2014). High-throughput sequencing for the study of bacterial pathogen biology. Curr. Opin. Microbiol..

[49] Noble S.M., French S., Kohn L.A., Chen V., Johnson A.D. (2010). Systematic screens of a *Candida albicans* homozygous deletion library decouple morphogenetic switching and pathogenicity. Nat. Genet..

[50] Liu O.W., Chun C.D., Chow E.D., Chen C., Madhani H.D., Noble S.M. (2008). Systematic genetic analysis of virulence in the human fungal pathogen *Cryptococcus neoformans*. Cell.

[51] Sasse A., Hamer S.N., Amich J., Binder J., Krappmann S. (2016). Mutant characterization and in vivo conditional repression identify aromatic amino acid biosynthesis to be essential for *Aspergillus fumigatus* virulence. Virulence.

[52] Phadke S.S., Maclean C.J., Zhao S.Y., Mueller E.A., Michelotti L.A., Norman K.L., Kumar A., James T.Y. (2018). Genome-wide screen for *Saccharomyces cerevisiae* genes contributing to opportunistic pathogenicity in an invertebrate model host. G3.

[53] Seed K.D., Dennis J.J. (2009). Experimental bacteriophage therapy increases survival of *Galleria mellonella* larvae infected with clinically relevant strains of the *Burkholderia cepacia* complex. Antimicrob. Agents Chemother..

[54] Deacon J., Abdelghany S.M., Quinn D.J., Schmid D., Megaw J., Donnelly R.F., Jones D.S., Kissenpfennig A., Elborn J.S., Gilmore B.F. (2015). Antimicrobial efficacy of tobramycin polymeric nanoparticles for *Pseudomonas aeruginosa* infections in cystic fibrosis: Formulation, characterisation and functionalisation with dornase alfa (DNase). J. Control. Release.

[55] Kamal F., Dennis J.J. (2015). *Burkholderia cepacia* complex Phage-Antibiotic Synergy (PAS): Antibiotics stimulate lytic phage activity. Appl. Environ. Microbiol..

[56] Ross-Gillespie A., Weigert M., Brown S.P., Kummerli R. (2014). Gallium-mediated siderophore quenching as an evolutionarily robust antibacterial treatment. Evol. Med. Public Health.

[57] Antunes L.C., Imperi F., Minandri F., Visca P. (2012). In vitro and in vivo antimicrobial activities of gallium nitrate against multidrug-resistant Acinetobacter baumannii. Antimicrob. Agents Chemother..

[58] Coughlan A., Scanlon K., Mahon B.P., Towler M.R. (2010). Zinc and silver glass polyalkenoate cements: An evaluation of their antibacterial nature. Biomed. Mater. Eng..

[59] Browne N., Hackenberg F., Streciwilk W., Tacke M., Kavanagh K. (2014). Assessment of in vivo antimicrobial activity of the carbene silver(I) acetate derivative SBC3 using *Galleria mellonella* larvae. Biometals.

[60] Rowan R., Moran C., McCann M., Kavanagh K. (2009). Use of *Galleria mellonella* larvae to evaluate the in vivo anti-fungal activity of [Ag2(mal)(phen)3]. Biometals.

[61] Gibreel T.M., Upton M. (2013). Synthetic epidermicin NI01 can protect *Galleria mellonella* larvae from infection with *Staphylococcus aureus*. J. Antimicrob. Chemother..

[62] Dean S.N., Bishop B.M., van Hoek M.L. (2011). Susceptibility of *Pseudomonas aeruginosa* biofilm to α-helical peptides: D-enantiomer of LL-37. Front. Microbiol..

[63] Chibebe Junior J., Fuchs B.B., Sabino C.P., Junqueira J.C., Jorge A.O., Ribeiro M.S., Gilmore M.S., Rice L.B., Tegos G.P., Hamblin M.R. (2013). Photodynamic and antibiotic therapy impair the pathogenesis of Enterococcus faecium in a whole animal insect model. PLoS ONE.

[64] Latz S., Kruttgen A., Hafner H., Buhl E.M., Ritter K., Horz H.P. (2017). Differential effect of newly isolated phages belonging to PB1-Like, phiKZ-Like and LUZ24-Like Viruses against Multi-Drug Resistant *Pseudomonas aeruginosa* under varying growth conditions. Viruses.

[65] Koch G., Nadal-Jimenez P., Reis C.R., Muntendam R., Bokhove M., Melillo E., Dijkstra B.W., Cool R.H., Quax W.J. (2014). Reducing virulence of the human pathogen *Burkholderia* by altering the substrate specificity of the quorum-quenching acylase PvdQ. Proc. Natl. Acad. Sci. USA.

[66] Bastidas R.J., Shertz C.A., Lee S.C., Heitman J., Cardenas M.E. (2012). Rapamycin exerts antifungal activity in vitro and in vivo against *Mucor circinelloides* via FKBP12-dependent inhibition of Tor. Eukaryot. Cell.

[67] Blatzer M., Blum G., Jukic E., Posch W., Gruber P., Nagl M., Binder U., Maurer E., Sarg B., Lindner H. (2015). Blocking Hsp70 enhances the efficiency of Amphotericin B treatment in resistant *Aspergillus terreus* strains. Antimicrob. Agents Chemother..

[68] Favre-Godal Q., Dorsaz S., Queiroz E.F., Conan C., Marcourt L., Wardojo B.P., Voinesco F., Buchwalder A., Gindro K., Sanglard D. (2014). Comprehensive approach for the detection of antifungal compounds using a susceptible strain of *Candida albicans* and confirmation of in vivo activity with the *Galleria mellonella* model. Phytochemistry.

[69] Cowen L.E., Singh S.D., Kohler J.R., Collins C., Zaas A.K., Schell W.A., Aziz H., Mylonakis E., Perfect J.R., Whitesell L. (2009). Harnessing Hsp90 function as a powerful, broadly effective therapeutic strategy for fungal infectious disease. Proc. Natl. Acad. Sci. USA.

[70] Fuchs B.B., Li Y., Li D., Johnston T., Hendricks G., Li G., Rajamuthiah R., Mylonakis E. (2016). Micafungin elicits an immunomodulatory effect in *Galleria mellonella* and mice. Mycopathologia.

[71] Allegra E., Titball R.W., Carter J., Champion O.L. (2018). *Galleria mellonella* larvae allow the discrimination of toxic and non-toxic chemicals. Chemosphere.

[72] Thomas R.J., Hamblin K.A., Armstrong S.J., Muller C.M., Bokori-Brown M., Goldman S., Atkins H.S., Titball R.W. (2013). *Galleria mellonella* as a model system to test the pharmacokinetics and efficacy of antibiotics against *Burkholderia pseudomallei*. Int. J. Antimicrob. Agents.

[73] Adamson D.H., Krikstopaityte V., Coote P.J. (2015). Enhanced efficacy of putative efflux pump inhibitor/antibiotic combination treatments versus MDR strains of Pseudomonas aeruginosa in a *Galleria mellonella* in vivo infection model. J. Antimicrob. Chemother..

[74] Lange A., Beier S., Huson D.H., Parusel R., Iglauer F., Frick J.S. (2018). Genome sequence of *Galleria mellonella* (Greater Wax Moth). Genome Announc..

[75] Vogel H., Altincicek B., Glockner G., Vilcinskas A. (2011). A comprehensive transcriptome and immune-gene repertoire of the lepidopteran model host *Galleria mellonella*. BMC Genom..

